# Evaluation of *mrk*D, *pga*C and *wca*J as biomarkers for rapid identification of *K. pneumoniae* biofilm infections from endotracheal aspirates and bronchoalveolar lavage

**DOI:** 10.1038/s41598-024-69232-7

**Published:** 2024-10-09

**Authors:** Naveen Kumar Devanga Ragupathi, Dhiviya Prabaa Muthuirulandi Sethuvel, Anju Ganesan, Dhivya Murugan, Ashtawarthani Baskaran, Dhammika Leshan Wannigama, Peter N. Monk, Esther Karunakaran, Balaji Veeraraghavan

**Affiliations:** 1 Department of Chemical and Biological Engineering, The University of Sheffield, Sheffield, S1 3JD UK; 2 Biofilms and Antimicrobial Resistance Consortium of ODA Receiving Countries (BARCOD), The University of Sheffield, Sheffield, UK; 3 Department of Clinical Microbiology, Christian Medical College, Vellore, India; 4 Department of Microbiology, Faculty of Medicine, Chulalongkorn University, King Chulalongkorn Memorial Hospital, Thai Red Cross Society, 1873 Rama 4 Road, Pathumwan, Bangkok, 10330 Thailand; 5 School of Medicine, Faculty of Health and Medical Sciences, The University of Western Australia, Nedlands, WA Australia; 6 Pathogen Hunter’s Research Collaborative Team, Department of Infectious Diseases and Infection Control, Yamagata Prefectural Central Hospital, Yamagata, Japan; 7 Department of Infection, Immunity and Cardiovascular Disease, The University of Sheffield, Sheffield, UK

**Keywords:** Biofilms, Diagnosis, Antimicrobial resistance, Biomarker, Nosocomial, *K. pneumoniae*, Diagnostic markers, Infectious diseases, Bacteria, Biofilms

## Abstract

*Klebsiella pneumoniae* has been identified as one of the most important opportunistic pathogens responsible for nosocomial infections. Antibiotic resistance and the ability to form biofilms are the two main factors involved in the persistence of infections. Conventional detection methods involve culture isolation and identification followed by biofilm assay that takes 48–72 h. Timely detection of biofilm-forming resistant pathogens is essential to appropriately treat the infection with the right dose and combinations. The present study focuses on evaluating an RT-PCR panel using *mrk*D, *pga*C, and *wca*J genes to screen for biofilm-forming *K. pneumoniae* from ETA/BAL specimens. The assay accurately identified *K. pneumoniae* harboring samples with a limit of detection of 1 ng/µl total RNA. Representative culture-negative-PCR-positive samples were subjected to metagenomics which identified *K. pneumoniae* reads in these samples confirming the specificity of RT-PCR. *mrk*D and *pga*C act as *K. pneumoniae* specific identification whereas *wca*J acts as a negative marker for biofilm-forming *K. pneumoniae*. In addition, RT-PCR results correlated well with the phenotypic biofilm-forming assay. This RT-PCR assay is the first of its kind for rapid identification of biofilm-forming *K. pneumoniae*. The result of this study highlights that the rapid detection of *K. pneumoniae* biofilms based on the RT-PCR results coupled with clinical conditions would be appropriate to treat emerging infections or to prevent re-infections in clinical settings.

## Introduction

*Klebsiella pneumoniae* is an opportunistic Gram-negative pathogen frequently implicated in catheter-associated and urinary tract infections. In hospitalized patients, intestinal *K. pneumoniae* carriage was substantially linked to recurrent infections^1^. Similarly, colonization of the oropharynx by *K. pneumoniae* is linked to a higher risk of *K. pneumoniae* ventilator-associated pneumonia (VAP)^2^. This has become a serious threat worldwide due to the spread of hypervirulent and antibiotic resistance strains presenting high mortality and morbidity rates^3^.

*K. pneumoniae* is one of the important nosocomial pathogens with the potential to form biofilms in vitro and in vivo and is clinically significant in patients associated with medical devices such as catheters, endotracheal tubes, and artificial implants. *K. pneumoniae* biofilms developed on solid surfaces promote cell adherence, formation of microcolonies, maturation, and finally dispersal as free-living cells. Capsular polysaccharides and fimbriae (type 3) are vital in forming biofilm structures^4^. Fimbriae maintain stable adherence, whereas capsular polysaccharides influence communication between cells and the biofilm structure. *K. pneumoniae* is protected by its biofilm structure from the host immune response and antibiotic penetration thereby contributing to drug resistance^5^.

The clinical *K. pneumoniae* biofilm formation mechanism is associated with a series of genes including allantoin (*allS*), capsular polysaccharide (CPS) (*treC*, *cpsD*, *wzc*, *wabG*, *wcaG*, *rmpA*/*A2*, *wzyk2* and *magA*), aerobactin (*iutA*), polysaccharides and adhesins (*pgaA*, *pgaB*, *pgaC*, and *bcsA*), type 1 (*fimA* and *fimH*) and type 3 fimbriae (*mrkD* and *mrkA*), quorum sensing (QS) (*luxS*) and colonic acid^6–11^.

Eradication of the biofilm cells is challenging when compared with planktonic cells. *K. pneumoniae* biofilms are highly resistant to almost all commonly used antibiotics^12^. Antimicrobial susceptibility testing (AST) is difficult in biofilm situations^13^. Biofilms and the analysis of the increased requirement of drug concentrations for their eradication need special methods and expertise. However, limited data are available on the effectiveness of ceftazidime/avibactam, aztreonam, and colistin against biofilm infections, unlike beta-lactams having poor penetration capacity in biofilm structures^14^.

The rapid detection method of *K. pneumoniae* biofilm can contribute to early diagnosis and adopt appropriate treatment regimens to improve disease management. Specific biomarkers to screen for *K. pneumoniae* biofilms using the real-time (RT) PCR method will ensure early detection with utmost sensitivity and specificity. Timely detection of biofilm-forming pathogens is essential for appropriate treatment with the right dose and combinations. The present study focuses on evaluating markers *mrk*D, *pga*C, and *wca*J to screen for biofilm-forming *K. pneumoniae* from clinical specimens.

## Results

Among 351 isolates of *K. pneumoniae* from patients with blood-stream infection, 65% were susceptible to minocycline, followed by 60% susceptibility to carbapenem and chloramphenicol. *K. pneumoniae* isolates susceptible to amikacin and netilmicin were found to be 59%. Whereas, other tested antimicrobials showed < 55% susceptibility including cefoperazone/sulbactam 55%, piperacillin/tazobactam 50%, tigecycline 50%, tetracycline 48%, cephalosporins ~ 40%, and ciprofloxacin 31%.

Among 114 isolates of *K. pneumoniae* tested from patients with respiratory infections [Endotracheal Aspirate (ETA) / Bronchoalveolar Lavage (BAL)], 89% were susceptible to gentamicin followed by 47% and 46% to tobramycin and carbapenems, respectively. Whereas, other tested antimicrobials exhibited < 45% susceptibility including levofloxacin 44%, cefoperazone/sulbactam 43%, piperacillin/tazobactam 36%, and cephalosporins ~ 35%.

For further analysis, 33 ETA, 9 BAL samples, and 66 clinical K. pneumoniae isolates (blood and ETA/BAL) were included. As evaluated by RT-PCR, *mrkD, pgaC,* and *wcaJ* genes exhibited a decrease in Ct values with increasing RNA concentration (Fig. S1).

### Biofilm forming capacity

**Figure 1:**
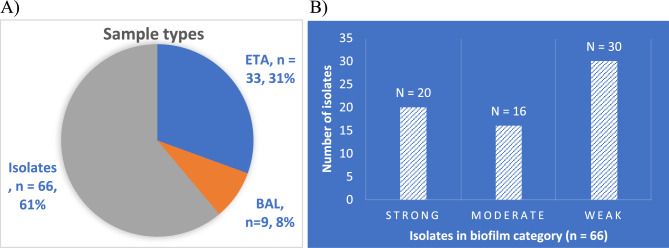
Distribution of sample types, Isolates (N = 66), ETA (N = 33) and BAL (N = 9) included in the study (**A**) and biofilm-forming capacity of the *K. pneumoniae* clinical strains (**B**).

*K. pneumoniae* isolates were subjected to biofilm formation using a microtiter plate assay with crystal violet staining and quantification. Out of 66 isolates screened for biofilm formation, 20 K*. pneumoniae* isolates were strong biofilm producers, 16 were moderate biofilm producers and 30 were weak biofilm producers (Fig. 1).

### RT-PCR for identification of biofilm-forming *K. pneumoniae*

The expression of *mrk*D and *pga*C were observed with their low Ct values in biofilm-forming *K. pneumoniae* isolates (*n* = 34) and the standard strain used as positive control (PC). Whereas, *P. aeruginosa* used as negative control (NC) was undetermined or ≥ 30 Ct for *mrk*D and > 30 for *pga*C genes, respectively. The RT-PCR interpretative criteria for biofilm/non-biofilm *K. pneumoniae* are shown in Table 1. Accordingly, presence of *wca*J indicates non-biofilm-forming *K. pneumoniae*, whereas *wca*J negative and *mrk*D positive irrespective of *pga*C indicates biofilm-forming *K. pneumoniae*.

**Table 1:** Interpretative criteria for *K. pneumoniae* RT-PCR from direct sample.

	*mrk*D	*pga*C	*wca*J
Biofilm forming	+	+ /−	−
Non-biofilm forming	+ /−	+ /−	+
Non-Klebsiella	−	−	−

Using RT-PCR, all clinical *K. pneumoniae* isolates were positive for *mrk*D and *pga*C, while borderline for *wca*J. This showed 100% sensitivity and specificity in comparison to conventional microbiological tests (CMT) for identification of *K. pneumoniae*. Further, the ct value of marker genes and the biofilm-forming ability of isolates exhibited a good correlation. Briefly, all strong biofilm-forming *K. pneumoniae* expressed the *mrk*D gene with a ct value of < 15 whereas the moderate and weak biofilm formers showed a ct range between 17–22 and 18–25 respectively. Similarly, for *pga*C gene, ct range of 13–18 was observed for strong biofilm-forming *K. pneumoniae*, whereas 19–23 and 20–25 for moderate and weak biofilm formers respectively. Overall, significantly low ct values were observed for strong biofilm formers compared to other categories, indicating higher expression of the marker genes.

### RT-PCR based identification of *K. pneumoniae* from direct samples

For evaluation, 76 ETA/BAL samples were subjected to RT-PCR assay. Of these, 44 were culture positive and 32 were culture negative for *K. pneumoniae*. All culture-positive samples were also positive for marker genes *mrk*D and *pga*C in RT-PCR. However, 20 culture-negative samples were also detected as *K. pneumoniae* positives by RT-PCR. The sensitivity and specificity calculated compared to the culture method as the gold standard is given in Table 2. The sensitivity of the assay to detect *K. pneumoniae* in samples has a limit of detection as low as 1 ng/µl total RNA (Fig. S1).

**Table 2:** Sensitivity and specificity of RT-PCR to detect *K. pneumoniae* from direct patient samples.

N = 76	Culture + ve (*n* = 44)	Culture -ve (*n* = 32)	
RT-PCR + ve (*n* = 64)	44 (TP)	20 (FP)	PPV = TP/(TP/FP) = 44/64 = 68.75%
RT-PCR -ve (*n* = 12)	0 (FN)	12 (TN)	NPV = TN/(TN + FN) = 12/(12) = 100%
	Sensitivity – TP/(TP + FN) = 100%	Specificity – TN/(TN + FP) = 12/32 = 37.5%	

*TP* True positives, *TN* True negatives, *FP* False positives, *FN* False negatives, *PPV* Positive predictive value, *NPV* Negative predictive value.

### Metagenomics of direct samples

A total of 6 ETA and 2 BAL samples were included for metagenome sequencing. Of these, 5 ETA and 2 BAL samples were positive for *K. pneumoniae* by RT-PCR. 16S rRNA metagenomic sequencing revealed the presence of *Klebsiella* reads in all these RT-PCR-positive samples. Of these 8 samples, four were culture positive and four were culture negative for *K. pneumoniae*. All culture positives were also positive by RT-PCR and metagenomics. Whereas, among four culture negatives, three were positive by RT-PCR and metagenomics. The remaining culture-negative sample, SP2545 was confirmed to be truly negative by both RT-PCR and metagenomics (Table 3). This sample, SP2545 did not harbour any *K. pneumoniae* isolates as identified by the standard culture method, and was negative by RT-PCR for all three genes tested, *mrk*D, *pga*C, and *wca*J. For confirmation, the 16S metagenome of this sample was performed which also turned negative for *K. pneumoniae*.

**Table 3:** Confirmation of culture-negative vs RT-PCR positives using 16S metagenomics.

Sample id	Sample type	Organism identified in culture	*mrk*D	*pga*C	*wca*J	Metagenome KPN Reads
SP1385	ETA	*NFGNB, B. cenocepacia, C. albicans/glabrata*	+	+	−	Present
SP 1475	BAL	*Normal flora*	+	−	−	Present
SP 1487	ETA	*Stenotrophomonas maltophilia, Acinetobacter baumannii*	+	−	−	Present
SP 2545	ETA	*ABC, NFGNB (burkholderia)*	−	−	−	Absent
SP 1460	ETA	*Stenotrophomonas maltophilia, Acinetobacter baumannii, Klebsiella pneumoniae*	+	+	−	Present
SP 2438	ETA	*Klebsiella, Escherichia coli*	+	+	−	Present
SP 3804	BAL	*Klebsiella, Escherichia coli*	+	+	−	Present
SP 1401	ETA	*Pseudomonas aeruginosa, Klebsiella pneumoniae*	+	+	−	Present

+ is < 30 Ct values; − is ≥ 30 Ct values by RT-PCR; KPN – *K. pneumoniae.*

**Figure 2:**
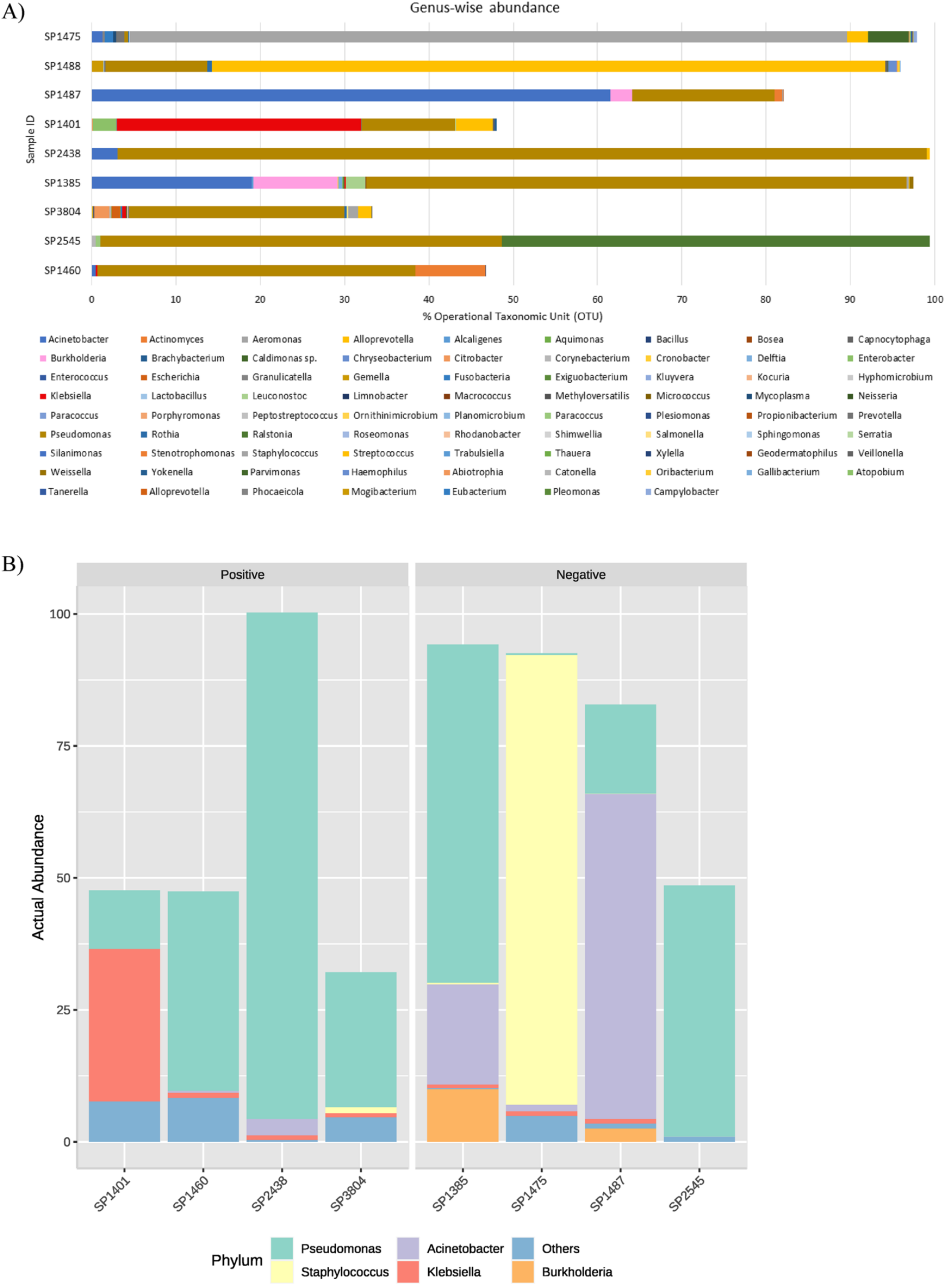
Metagenomic data revealing percentage OTU abundance for genus-wise distribution of reads among metagenome-sequenced ETA/BAL samples (**A**) Graph showing highly abundant genera between culture positive and negative group (**B**).

Figure 2A depicts the abundance of various genera identified among the eight sequenced samples, where *Klebsiella* reads from culture positive and negative groups correlate with the RT-PCR results (Fig. 2B). Results of the culture-negative RT-PCR-positive group confirm the sensitivity of RT-PCR assay to be 100% for identification of *K. pneumoniae*.

## Discussion

Antimicrobial resistance is an increasing challenge in healthcare-associated infections. Multidrug-resistant *K. pneumoniae* is one of the main causes of nosocomial infections, especially among immunocompromised individuals. Drug resistance and biofilm-forming ability are the key virulence factors contributing to the persistence of infections^15^. The ability to produce biofilm was considerably higher in extensively drug-resistant (XDR) *K. pneumoniae* isolates (91.07%) than in MDR and susceptible strains, suggesting a positive link between antibiotic resistance profile and biofilm-forming ability^16^. Notably, in our previous study, it was identified that 90% of strong biofilm-forming *K. pneumoniae* were resistant to carbapenem, significantly higher than the weak and negative biofilm-forming *K. pneumoniae*^17^. Increasing AMR in *K. pneumoniae* has become a worldwide problem, and there is still limited data on biofilm-producing *K. pneumoniae* in India. These biofilm-forming *K. pneumoniae* are highly resistant to many commonly used antibiotics thus making the current treatment challenging.

The situation becomes more complicated when biofilm-producing organisms are treated with inappropriate antibiotics with insufficient concentrations. For instance, biofilms can resist empiric antibiotic therapy and contribute to bacterial persistence, making this challenging infection more severe^18^. Antibiotics such as piperacillin, piperacillin/tazobactam, cefoperazone, ceftazidime, cefepime, meropenem, ciprofloxacin, netilmicin, and amikacin were reported to show reduced activity against adherent bacteria when compared to the planktonic counterparts^19^. In addition, several studies have shown that certain antibiotics induce biofilm formation when treated with sub-inhibitory concentration^20^. For this reason, early detection of biofilm-forming nosocomial pathogens mainly from critical-care units is crucial. The present study was designed to inform clinicians in identifying and choosing the appropriate antibiotic therapy for biofilm-mediated infections, which require combination therapy or higher class antibiotic therapy rather than standard empirical therapy.

The key aspect of this study was to develop a diagnostic assay that uses a set of virulence genes of *K. pneumoniae* as a marker. Currently, no rapid diagnostic methods are available to identify *K. pneumoniae* biofilms from clinical isolates and direct samples. Conventional assays take 48–72 h for identification of *K. pneumoniae* and biofilm formation ability. This assay based on real-time (RT)-PCR provided an added advantage by offering information on the biofilm-forming ability of *K. pneumoniae* with pathogen identity in less than eight hours.

Among the various genes responsible for biofilm production, *mrk* (Type 3 fimbriae) and *pga*C (polysaccharide adhesion) are the candidate genes linked with biofilm formation in *K. pneumoniae* and have shown to promote strong biofilm formation, enabling surface adhesion^17^. Studies have indicated that the *mrkA* gene contributes to rapid biofilm formation while *mrkD* was responsible for dense *K. pneumoniae* biofilms^21–24^. MrkD, a fimbrial adhesin from *Klebsiella pneumoniae*, causes adherence to the basement membranes of tissues and the basolateral surfaces of renal and pulmonary epithelia. This adhesin, an extracellular matrix-binding protein, has been demonstrated to bind to type V collagen. Even though all isolates containing the MrkD adhesin induce the agglutination of erythrocytes treated with tannic acid *in-vitro*, the *mrk*D gene is not conserved across species. The ability of a plasmid-borne *mrk*D gene product to induce type V collagen binding is usually associated with *K. oxytoca* strains and seldom with *K. pneumoniae* strains. The MrkD adhesin is a chromosomally borne adhesin that mediates binding to collagen type IV and V in *K. pneumoniae*^25,26^*.*

Similarly, *pga*C also reported to serve as an adhesion factor for the initiation and maintenance of biofilm structure^27^. *pga*C is known to be closely associated with *pga*B/D, a biofilm adhesin polysaccharide, and *lux*R gene, an *N*-acyl homoserine lactone (AHL)-dependent transcriptional regulator. AHL is one of the most common quorum sensing (QS) mechanisms utilized by Proteobacteria^28^. QS is a well-established mechanism in the process of biofilm formation^29^. LuxR protein plays a key role in the QS mechanism in most Gram-negative bacteria by detecting the presence of signaling molecules that enable inter- and intra-species interaction in response to external stimuli according to population density^30^.

Furthermore, *in-silico* screening of 451 clinical *K. pneumoniae* genomes available in the public database revealed that 98%, 99.3% and 34.6% of the genomes carried *mrk*D, *pga*C, and *wca*J genes^17^. It was also found that *mrk*D and *pga*C genes were present in all biofilm-forming *K. pneumoniae* isolates in the present study. This indicates their conservation in *K. pneumoniae* strains and the specificity of the chosen targets in the detection of *K. pneumoniae* biofilms from clinical samples.

Occasionally, some strains of *K. pneumoniae* may lose their fimbriae during culturing or may lack the fimbriae gene^24^. This issue has been addressed by using more than one target as demonstrated in the present study to ensure the reliability of the assay. Interestingly, the PCR panel evaluated in this study identified *K. pneumoniae* from even the culture-negative samples, showing the high sensitivity of the assay. Further, the samples that were PCR positive and culture negative were confirmed by 16S metagenomics to have *Klebsiella* reads. This could be due to the limited sensitivity of the culture method, or the lesser load of the pathogen in the sample. In such cases, treatment with broad-spectrum antibiotics will be helpful since *Klebsiella* in this individual may or may not be associated with the active infection due to its low bacterial load. However, this approach also has its limitations where the patient may be over-treated leading to the development of drug resistance. To avoid this difficulty, the RT-PCR results can be coupled with clinical diagnosis to make this an accurate diagnostic tool.

Further, the *wca*J gene appears to act as a negative regulator, where its absence indicates the high potential of the biofilm-forming capacity of the strain. The hypothesis was supported by an earlier study by Pal et al., where they demonstrated that the inactivation of the *wcaJ* gene results in the disruption of colanic acid synthesis and enhances the biofilm formation in *K. pneumoniae*^7^. Based on the observed results for known positives and negatives, the cut-off for Ct values to define negatives was ≥ 30 Ct for *mrk*D and > 30 for *pga*C genes. However, this needs further standardization with a higher number of clinical samples.

Antimicrobial resistance rates among biofilm-forming bacteria are higher compared to its planktonic forms and above the breakpoints proposed for therapeutic clinical use. This shows that treatment of biofilms with standard antimicrobial therapy would be unhelpful mainly among patients in high-dependency units. This may also explain the treatment failure in some patients, despite susceptibility to antimicrobials in vitro, resulting in clinical resistance. The PCR evaluated in this study in combination with clinical diagnosis will help in the early detection of *K. pneumoniae* biofilms in critically ill patients and for their appropriate treatment either with high-dosage broad-spectrum antimicrobials or with combinations.

In conclusion, the PCR assay standardized in this study is the first of its kind for rapid identification of biofilm-forming *K. pneumoniae* from clinical samples. Considering the limited resource settings like primary health laboratories, the cost of the PCR test and maintenance of the sample integrity might be the limiting factors. Overall, the results of the study highlight that the rapid detection of *K. pneumoniae* biofilms based on the real-time PCR results coupled with clinical conditions would be appropriate to treat emerging infections or to prevent re-infections in clinical settings.

## Materials and methods

### Study samples

The study includes direct respiratory samples (ETA/BAL) received for routine bacteriological culture at the Department of Clinical Microbiology from patients admitted to intensive care units at Christian Medical College, Vellore, India. For this study, samples were used after the routine processing for which it was collected. *K. pneumoniae* isolates obtained from these respiratory samples were also included for the evaluation of the rapid screening assay.

### Antimicrobial susceptibility testing

Antimicrobial susceptibility testing was performed by the Kirby-Bauer disc diffusion method against cefotaxime (30 μg), ceftazidime (30 μg), cefuroxime (30 μg), cefepime (30 μg), piperacillin-tazobactam (100/10 μg), cefoperazone-sulbactam (75/30 μg), ciprofloxacin (5 μg), levofloxacin (5 μg), trimethoprim-sulfamethoxazole (75/30 μg), tetracycline (30 μg), meropenem (10 μg), ertapenem (10 μg), amikacin (30 μg), gentamycin (10 μg), tobramycin (30 μg) and minocycline (30 μg) according to CLSI, 2021 guidelines^31^. Quality control strains used were *Escherichia coli* ATCC 25922 for all antibiotics concurrently in all the batches. Tigecycline results were interpreted according to FDA criteria.

### Biofilm screening assay

The screening assay was performed as described previously by Devanga Ragupathi et al.^17^. Briefly, about 5–10 fresh colonies were inoculated into a 10 ml LB broth and incubated at 37 °C for 12–18 h. The optical density (OD) was measured in a spectrophotometer (Shimadzu, Kyoto, Japan) at 625 nm and 0.05 OD cells prepared by dilution in Mueller–Hinton broth (MHB) containing 1% glucose. OD-adjusted cells were inoculated into a 96-well plate and incubated at 37 °C for 24 h. After 24 h of incubation, the medium was removed, and the biofilm was washed with 200 μl of distilled water. The biofilm was later stained with 200 μl 0.1% (w / v) crystal violet dye and incubated for 10 min at RT. OD was read at 570 nm after de-stained with glacial acetic acid followed by 5 min incubation at RT. The assay was performed in triplicate. Broth without cells was used as a negative control. A well-characterized biofilm-forming *K. pneumoniae* strain (K355) was used as a positive control. The biofilm production was classified as OD < ODc = poor biofilm producer; ODc < OD ≤ 2 × ODc = weak biofilm producer; 2 × ODc < OD < 4 × ODc = moderate biofilm producer; and OD ≥ 4 × ODc = strong biofilm producer.

### RNA isolation using guanidinium isothiocyanate

Guanidinium Isothiocyanate (GITC) is a chaotropic agent that disrupts cells, denatures proteins, and deactivates nucleases, thereby stabilizing the nucleic acid. Briefly, a 10 µl loop full of bacteria was suspended in 400 µl of saline and 400 µl of GITC lysis buffer (4 M GITC, 25 mM Tris–HCl). The suspension was vortexed for 15 s then incubated at 56 °C for 15 min on the heating block. After that (96–100% ethanol) was added and vortexed for 15 s, after which the lysate was transferred to a spin column and incubated at RT for 5 min. After centrifugation at 6000 × g for 1 min, the supernatant was discarded. RNA was then washed with wash buffer I (0.9 M GITC, 10 mM Tris, 20% EtOH) and wash buffer II (100 mM NaCl, 10 mM Tris–HCl, 80% EtOH). After washing, the spin column dried at 56 °C for 3 min. Finally, RNA was eluted in fresh Eppendorf tubes by adding 50 µl of RNase-free water into the spin column followed by centrifugation at 6000 × g for 5 min. The quality and purity of the RNA obtained was evaluated using the Qubit and Nanodrop spectrophotometers. A well-characterized biofilm-forming *K. pneumoniae* strain (K355) was used as positive control and *P. aeruginosa* ATCC 27,853 as negative control for RT-PCR experiments.

### Reverse transcriptase PCR

cDNA was synthesized by Reverse transcriptase kit using 1 µl of the primer mix, 1 µl of Reverse Transcriptase (RT), 4 µl of RT Buffer 5X, and 14 µl of RNA template, and the reaction mixture was incubated at 42 °C for 15 min and then incubated at 95 °C for 3 min then the product is kept in ice while preparing reaction mixture for real-time PCR.

### Real-time PCR for detection of biofilm-forming *K. pneumoniae*

Real-time PCR 7500 Fast DX instrument was used for gene amplification and relative quantification. Amplifications were performed using the instrument’s programmed two-step real-time PCR. Table 4 shows the primers used for RT-PCR. The total reaction volume (20 μl) was prepared by mixing 10 μl of SYBR Green qPCR master mix, 1 μl of forward primer, 1 μl of reverse primer (2 pmol), 5 μl of template cDNA, and 3 μl of PCR water. The real-time PCR cyclic condition was programmed as follows; initial holding at 55 °C for 30 min and 94 °C for 2 min followed by 40 cycles of 94 °C for 15 s, 55 °C for 1 min, and 68 °C for 1 min.

**Table 4:** Primer sequences used for Real-Time PCR.

Gene	Forward Primer (5’ -3’)	Reverse Primer (5’ -3’)
*mrk*D	GCCACAACGCCTTACTGAAA	CTATTCTGCGCTGGTCATCG
*pga*C	ATGCCTGTTCCACGCTGTGG	CAGGCTTCCTTTTCCCCGGT
*wca*J	AAATGGCGTACCGGTTGTTC	CGGCCCTTTCGAGGTAGTTT

Real-time quantification of cDNA was carried out on an ABI 7500 PCR detection system (Applied Biosystems, UK) using the SYBR green PCR master mix. Real-time PCR was used to investigate the expression level of *mrk*D, *pga*C, and *wca*J genes measured by relative quantitation. The cycle of threshold (C_t_) was considered as the average threshold cycle number from three independent experiments.

### 16S metagenomic sequencing

#### Sampling and DNA extraction

A total of 6 ETA and 2 BAL samples received at the laboratory for routine testing were used for metagenomics assay. The collected samples were extracted by QIAmp DNA Mini Kit as per the manufacturer’s instruction with slight modifications in the sample preparation steps. Briefly, samples were incubated with lysozyme for 1 h and overnight lysis with ATL buffer and proteinase K. Following incubation, samples were added with 0.5% saponin at RT for 10 min and incubated for 1 h at 56 °C with NaCl. Lysed extracts were then transferred to spin columns for the purification of DNA.

#### Metagenome sequencing

Bacterial 16S hypervariable genes were amplified by PCR from DNA samples using a range of V2,4,8 and V3,6,7,9 oligonucleotide primers specific for domain bacteria using Ion 16S metagenomic kit (Life Technologies, USA). Following amplification, all the PCR products were quantified, end-repaired, ligated and nick-repaired by Ion Plus Fragment Library Kit (Life Technologies, USA). Emulsion PCR was carried out using the Ion OneTouch Hi-Q View kit (Life Technologies), and the samples were adjusted to a final concentration of 100 pM. Templated-ISPs were sequenced on 318-chip v_2_bc (2 Gb) micro-chip using the Ion Torrent Personal Genome Machine (Life Technologies, USA) for 850 flows.

#### Bioinformatic analysis

The raw reads obtained by sequencing were analyzed using QIIME2 pipeline. First, the raw sequences were demultiplexed and then denoised to filter out too short sequences, singletons and chimeras. Reads were compared with Curated MicroSEQ(R) 16S Reference Library v2013.1 and Curated Greengenes v13.5 to define genera with 97% similarity, and species with 99% similarity. Percentage OTU similarity between samples was analyzed in SPSS and plotted using Microsoft Excel.

### Data analysis

All statistical analysis was conducted using SPSS v and Microsoft Excel v. Sensitivity and Specificity of the RT-PCR assay was calculated in comparison with the culture method as gold standard using a conventional 2 × 2 table. The slope of concentration-dependent decrease in Ct in RT-PCR was calculated and R2 represents the coefficient of determination.

### Ethics approval

The study was approved by the Institutional Review Board and Ethical Committee, Christian Medical College, Vellore, India (IRB No.: 11940 dt 27-03-2019). The study was conducted according to the guidelines and protocols approved by the Institution.

### Informed consent

The requirement for informed consent from patients was waived by the Institutional Review Board and Ethical Committee of Christian Medical College, Vellore, India.

## Supplementary Information

Supplementary Figure S1.

## Data Availability

16S metagenomic raw data is available through SRA (NCBI BioProject PRJNA1060935).
