# Supplementary material for: Evaluation of mrkD, pgaC and wcaJ as biomarkers for rapid identification of K. pneumoniae biofilm infections from endotracheal aspirates and bronchoalveolar lavage
Source: Sci Rep. 2024 Oct 9;14:23572. doi: 10.1038/s41598-024-69232-7 (PMC11464835; doi:10.1038/s41598-024-69232-7)
Supplement: Supplementary file 1 — Supplementary Figure S1. [file 41598_2024_69232_MOESM1_ESM.docx]

**Supplementary Figure**

**Figure S1:** RNA concentration vs observed RT-PCR Ct values for *mrk*D, *pga*C and *wca*J genes from *K. pneumoniae*. Picture depicts decreased Ct values with increasing RNA concentration exhibiting the sensitivity of the RT-PCR with as low as 1 ng/µl RNA concentrations.
